# Association of Cognitive Impairment with Reduced Health-Related Quality of Life and Depression Among Survivors of Thrombotic Thrombocytopenic Purpura

**DOI:** 10.3390/hematolrep17050051

**Published:** 2025-09-27

**Authors:** Sruthi Selvakumar, Jia Yu, Jacob Meade, Shruti Chaturvedi

**Affiliations:** 1 Department of Medicine, Morsani College of Medicine, University of South Florida, Tampa, FL 33612, USA; 2 School of Medicine, University of Pittsburgh, Pittsburgh, PA 15261, USA; yu.jia@medstudent.pitt.edu; 3 Division of Hematology, Department of Medicine, Johns Hopkins University School of Medicine, Baltimore, MD 20125, USA; jmeade6@jhmi.edu

**Keywords:** thrombotic thrombocytopenic purpura, health-related quality of life, depression, neurocognitive function

## Abstract

**Background**: Immune-mediated thrombotic thrombocytopenic purpura (iTTP) survivors exhibit increased rates of psychological comorbidities, cognitive impairment (CI), and reduced health-related quality of life (HRQoL). This cross-sectional study investigated the prevalence of CI and its association with reduced HRQoL and depression among iTTP survivors. **Methods***:* iTTP survivors completed the Beck Depression Inventory (BDI-II), the SF-36 for evaluation of HRQoL, and the NIH Toolbox Cognition Battery. SF-36 scores and fluid cognition and crystallized cognition composite scores from the cognition battery were compared to the reference population. We examined associations of cognitive impairment with depression and HRQoL. **Results**: We enrolled 47 patients with iTTP; 76.6% were female, the median age was 51 (IQR 39, 60), and the median number of episodes was 2 (1, 3.5). Compared to the reference, iTTP survivors had significantly lower mean scores in seven SF-36 domains (physical function, physical limitation, general, mental health, vitality, social functioning, and emotional limitation) as well as the mental component score (MCS) (*p* < 0.0001) and physical component scores (PCS) (*p* < 0.0001). A lower physical HRQoL score was observed in those with mild (49.3 vs. 37.7, *p* = 0.005) and major (49.3 vs. 38.4, *p* = 0.007) CI compared to no CI. The fluid cognition composite score correlated strongly with the SF-36 Physical Component Summary (r = 0.548, *p* = 0.0002) but not the Mental Component Summary (r = 0.113, *p* = 0.489). **Conclusions**: Cognitive impairment in iTTP survivors is associated with reduced physical HRQoL. Identifying and addressing cognitive deficits in iTTP may improve HRQoL. Given that 40% of participants had depressive symptoms, which were associated with reduced mental HRQoL, iTTP survivors may also benefit from routine mental health screening t.

## 1. Introduction

Immune-mediated thrombotic thrombocytopenic purpura (iTTP) is a rare hematologic disorder characterized by episodes of microvascular thrombosis and ischemic organ damage. Timely treatment with plasma exchange and immunosuppression has reduced acute iTTP mortality from >90% to <10% [1]. Since most patients survive their acute iTTP episode, managing late complications and survivorship issues is an important clinical and research priority. iTTP was previously viewed as primarily an acute condition, and survivors were expected to return to their previous level of health except for the 30–50% risk of relapse [2]. However, recent studies suggest that iTTP survivors experience numerous adverse long-term health outcomes following recovery. These include shortened survival and increased rates of chronic morbidities such as hypertension [3], obesity [4], stroke [5], renal injury [6], and autoimmune disease [7].

iTTP survivors also experience reduced health-related quality of life (HRQoL) [8], neurocognitive impairment [9,10], depression [10,11], and post-traumatic stress disorder (PTSD) [12]. Cognitive impairment in iTTP survivors is associated with silent cerebral infarction, or ischemic lesions on brain imaging, occurring in the absence of overt neurodeficits. The factors contributing to reduced HRQoL and adverse neuropsychological outcomes are unclear and may be due to coexisting chronic illnesses such as autoimmune disease and stroke [8,10,11]. However, reduced HRQoL may also be associated with cognitive impairment or depression, as has been described in other chronic disorders [13,14,15,16]. We conducted this cross-sectional study to determine the relationship between cognitive impairment and its influence on depression and reduced HRQoL. We further explored whether reduced HRQoL is driven more by physical or mental components and if iTTP survivors experienced more cognitive and affective symptoms of depression versus more somatic symptoms.

## 2. Materials and Methods

### 2.1. Participants and Recruitment

Adult (>18 years) participants with iTTP who had been in hematological remission from iTTP for at least 3 months were recruited from the hematology practice at Johns Hopkins University School of Medicine from 7 July 2020 to 31 December 2022. The diagnosis of iTTP was confirmed by documentation of an acute episode of thrombotic microangiopathy characterized by anemia (hemoglobin < 10 g/dL), thrombocytopenia (platelet count < 150 × 10^9^/L), and ADAMTS13 activity <10% during the acute episode. Informed consent was obtained from all participants through an online form prior to survey administration. The institutional review board at Johns Hopkins University approved this study.

### 2.2. Data Collection Instruments

Data was collected using an online survey that included (i) baseline demographic and socioeconomic information and two self-administered, validated tools, (ii) the Beck Depression Inventory-II (BDI-II), and (iii) Short Form-36 (SF-36) for HRQoL. In-person research visits were suspended temporarily during the study period due to the COVID-19 pandemic. However, a subset of participants underwent cognitive testing using the NIH Toolbox Cognition Battery [17] during in-person research visits once these were permitted. Additional clinical information including details surrounding iTTP diagnosis (number of episodes, time since last episode), existing psychiatric diagnoses (depression, post-traumatic stress disorder, anxiety disorder, or any other mental health condition), and past medical diagnoses (stroke, chronic kidney disease, hypertension, diabetes, systemic lupus erythematosus (SLE), etc.) were abstracted from the electronic medical record. Comorbid conditions were recorded as present or absent only, and the severity of these comorbidities was not recorded during our data collection. All data were collected, stored, and managed in REDCap, a secure web-based data management application hosted locally at Johns Hopkins University. The survey instruments are described below.

*Beck Depression Inventory-II (BDI-II):* The BDI-II is an extensively validated, self-administered depression screening tool. It includes 21 items, each scored on a 4-point Likert scale ranging from 0 to 3, with higher scores indicating worse symptoms. The total BDI-II score was interpreted as follows: no/minimal depression = 0–13, mild depression = 14–19, moderate depression = 20–28, and severe depression 29–63. At a cutoff of 14 and above, various studies have reported that the BDI-II detects depression with a sensitivity of 87.7–92% and specificity of 74–83% [18]. Scores on BDI-II items 1–14 (sadness, pessimism, past failure, loss of pleasure, guilty feelings, punishment feelings, self-dislike, self-criticalness, suicidal ideation, crying, agitation, loss of interest, indecisiveness, worthlessness) are summed to calculate cognitive/affective symptom scores. Items 15–21 (loss of energy, sleep problems, irritability, appetite problems, concentration, fatigue, loss of interest in sex) are summed to calculate somatic symptom scores.

*Short-form 36 (SF-36):* The SF-36 is an extensively validated 36-item short-form health survey that measures HRQoL and is divided into two components (physical and mental), each with four sub-domains [19]. Physical domains include physical functioning, physical role limitations, pain, and general health. Mental domains include mental wellbeing, emotional role limitations, social functioning, and vitality. Composite physical component summary (PCS) and mental component summary (MCS) scores are also calculated. All raw scores were first converted to a 0–100 scale score, from which z-scores were derived. These z-scores were compared to normed data derived from the general U.S. population. The PCS and MCS were normed with a reference population mean of 50 and standard deviation of 10, respectively. SF-36 scores were norm-based using pooled U.S. reference data from the SF-36 Health Survey: Manual and Interpretation Guide. The reference data was derived from the 1990 National Survey of Functional Health Status of non-institutionalized, U.S. adult (≥18 years) population (N = 2474) [19].

*NIH Toolbox Cognition Battery:* The NIH Toolbox Cognition Battery is a comprehensive, validated set of neurobehavioral measurements administered on an iPad. The NIH Toolbox Cognition Battery tests multiple cognitive domains including executive function (dimensional change card sort test, flanker inhibitory control and attention test), processing speed (pattern comparison test), episodic memory (picture sequence memory test), working memory (list sort working memory test), language (oral reading recognition, picture vocabulary test). In addition to individual test scores, composite scores for overall cognitive function, fluid cognition, and crystallized cognition are also computed. We used T scores adjusted for age, sex, race, and educational attainment, where the normative mean T score is 50 with a standard deviation (SD) of 10. An advantage of the NIH Toolbox Cognition Battery is that normative data from large populations are available to provide age, sex, and education-matched controls. For this study, we used the fully corrected T scores for the crystallized and fluid cognition composite to evaluate associations with HRQoL. The *Diagnostic and Statistical Manual of Mental Disorders*, fifth edition (DSM-5), defined mild and major cognitive impairments as T scores that are 1 or 2 SDs below the mean (between the third and 16th percentiles) and >2 SDs below the mean (below the third percentile) on at least one test, respectively, and we used these definitions in our study.

### 2.3. Statistical Analysis

Data were summarized as counts and proportions for categorical variables and either as mean and standard deviation or median and interquartile range (IQR representing the 25th to 75th percentile) for continuous variables. BDI-II, SF-36, and NIH Toolbox scores were computed and interpreted as described above. Mean norm-based SF-36 scores in each domain were compared with the reference population means using a single-sample *t*-test. To compare our cohort with the reference sample (N = 2474) while accommodating unequal variances and markedly different group sizes, we used the Welch’s *t*-test. We used the independent samples T-test to compare SF-36 MCS and PCS scores between patients with and without a positive screen for depression (BDI-II score ≥ 14). Finally, we examined the association between SF-36 MCS and PCS scores with the fluid and crystallized cognition composite scores on the NIH Toolbox Cognition Battery. *p* < 0.05 was considered statistically significant. Data management and statistical analyses were performed using RedCap (Research Electronic Data Capture version 14), Microsoft Excel (Microsoft Corporation, Redmond, WA, USA), and R (R Foundation for Statistical Computing, Vienna. Austria).

## 3. Results

### 3.1. Study Population Characteristics

Forty-seven participants were enrolled and completed the SF-36 survey between 7 July 2020, and 31 December 2022. Forty-four of these participants also completed the BDI-II survey, and forty participants completed the NIH Toolbox Cognition Battery. The median age of the participants was 51 (IQR 39, 60) years, and 76.6% were female. Participants had a median of 2 (IQR 1, 3.5) iTTP episodes. Psychiatric comorbidities such as PTSD, depression, and anxiety were reported in 6.4%, 31.9%, and 21.3%, respectively. Comorbidities included hypertension (42.6%), obesity (23.4%), prior history of stroke (19.1%), hyperlipidemia (19.1%), and systemic lupus erythematosus (19.1%). Table 1 summarizes demographic and characteristics of the cohort.

### 3.2. Cognitive Function in iTTP Survivors

**Figure 1:**
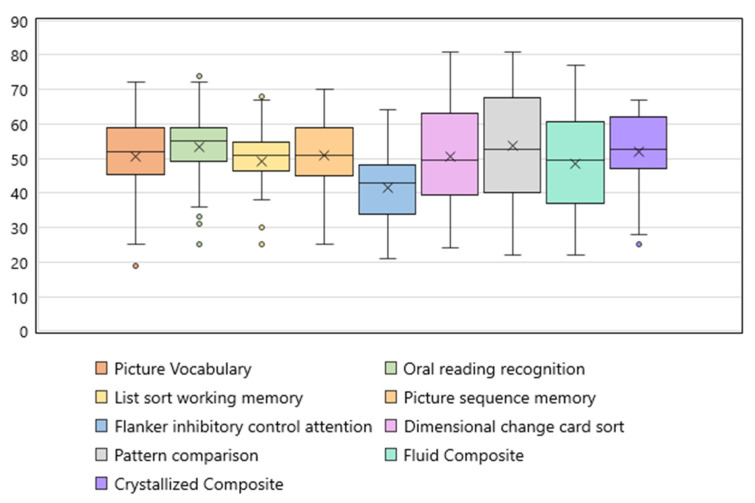
Components of NIH Toolbox Cognition Battery among survivors of iTTP (N = 40). The normative mean T score for each test is 50 with a standard deviation of 10. Scores < 40 and >30 are considered mild cognitive impairment, and scores ≤ 30 are considered major cognitive impairment.

The results of cognition testing on the NIH Toolbox Cognition Battery are shown in Figure 1. Of the forty patients that completed cognitive testing, twenty-one (53%) had mild cognitive impairment in at least one of seven domains (defined as a T score that is between 1–2 SD below the population mean, i.e., T scores < 40 and ≥30), including eleven participants with scores in at least one domain consistent with major cognitive impairment (T score < 2 SD below the mean, i.e., T score < 30).

### 3.3. Cognitive Impairment Is Associated with Reduced Health-Related Quality of Life in TTP Survivors

iTTP survivors had a significantly reduced physical health-related quality of life. iTTP survivors had lower mean scores than the reference population in all eight domains of HRQoL using the SF-36. This difference was statistically significant for seven domains including physical functioning (68.6 vs. 84.2, *p* < 0.0001), role limitations due to physical health (50.5 vs. 80.9, *p* < 0.0001), general health (50.2 vs. 71.9, *p* < 0.0001), mental health (65.3 vs. 74.7, *p* = 0.0004), vitality (46.3 vs. 60.9, *p* < 0.0001), social functioning (69.4 vs. 83.3, *p* < 0.0001) and role limitations due to emotional health (52.2 vs. 81.3, *p* < 0.0001) (Table 2). The mean physical component score (PCS 43.2, *p* < 0.0001) and mental component score (MCS 43.2, *p* < 0.0001) were also significantly lower than the reference population (reference mean, 50.0).

**Figure 2:**
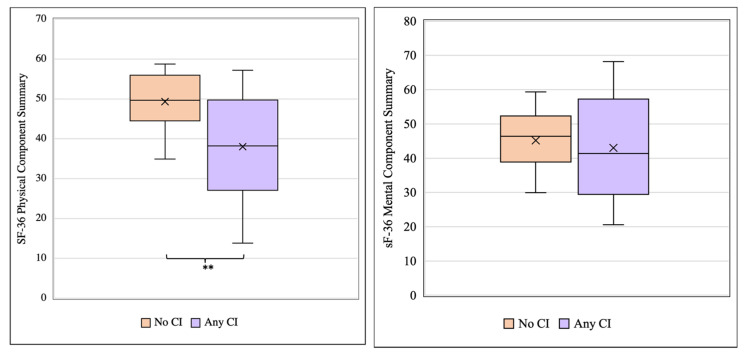
A comparison of SF-36 PCS and MCS scores among participants with no CI (n = 19) and any level of CI (n = 21). The ‘no CI’ group has significantly improved SF-36 PCS scores compared to the group with any degree of CI (49.3 vs. 38.0, *p *= 0.002). MCS scores did not differ significantly between the no CI vs. any CI groups (45.2 vs. 43.0, *p *= 0.588). ** indicates differences significant at *p* < 0.05.

**Figure 3:**
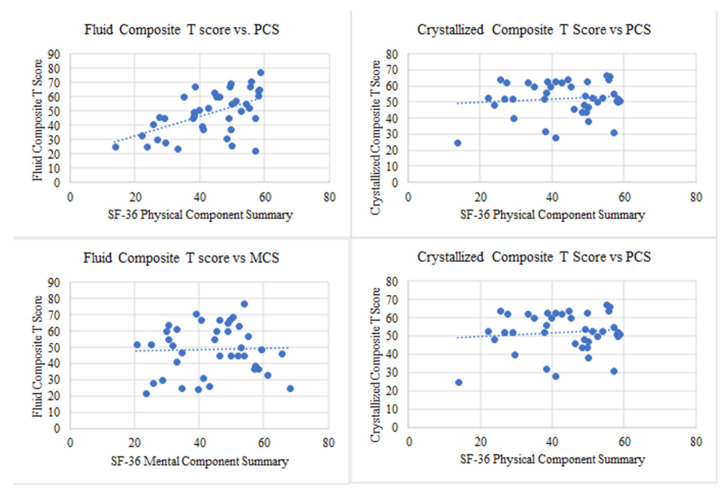
(**Top left**) A lower SF-36 PCS is associated with a lower fluid composite T-score with Pearson correlation r= 0.548 (*p *= 0.0002), suggesting that lower physical HRQoL may be linked with decreased fluid cognition. No significant correlation was found between PCS vs. crystallized composite score (r = 0.113, *p *= 0.489). (**bottom left**) No significant correlation was found between MCS and the fluid composite T-score (r = 0.037, *p *= 0.823) (**top right**) or the crystallized composite T-score (r = −0.080, *p *= 0.628) (**bottom right**).

Participants were categorized into one of three categories: no cognitive impairment (n = 19), mild cognitive impairment (n = 10), and major cognitive impairment (n = 11). The mean SF-36 PCS scores for those with no cognitive impairment were significantly higher compared to those with any level of cognitive impairment, which included the mild and major groups (49.3 vs. 38.0, *p *= 0.002) (Figure 2). Upon further analysis, both the mild cognitive impairment (49.3 vs. 37.7, *p *= 0.005) and major cognitive impairment (49.3 vs. 38.4, *p *= 0.007) groups had significantly lower mean PCS scores compared to the no cognitive impairment group, but no significant differences were observed between the mild and major cognitive impairment groups (37.7 vs. 38.4, *p *= 0.906) (Supplementary Figure S1). No significant differences in the mean SF-36 MCS scores were observed among the three cognitive impairment groups (Figure 3, Supplementary Figure S2).

The correlations between fluid cognition composite and crystallized cognition composite scores (T-scores; normed mean, 50 ± 10), which are adjusted for age, sex, education, and race, and mean MCS and PCS scores were also evaluated. SF-36 PCS scores were associated with fluid cognition composite scores (Pearson correlation coefficient, r = 0.548, *p *= 0.0002) but not with the crystallized cognition composite score (Pearson correlation coefficient, r = 0.113, *p *= 0.489) (Figure 3, top). There was no association between the MCS and the crystallized or fluid cognition composite scores (Figure 3, bottom).

### 3.4. iTTP Survivors with Cognitive Impairment Have an Increase in Depressive Symptoms

**Figure 4:**
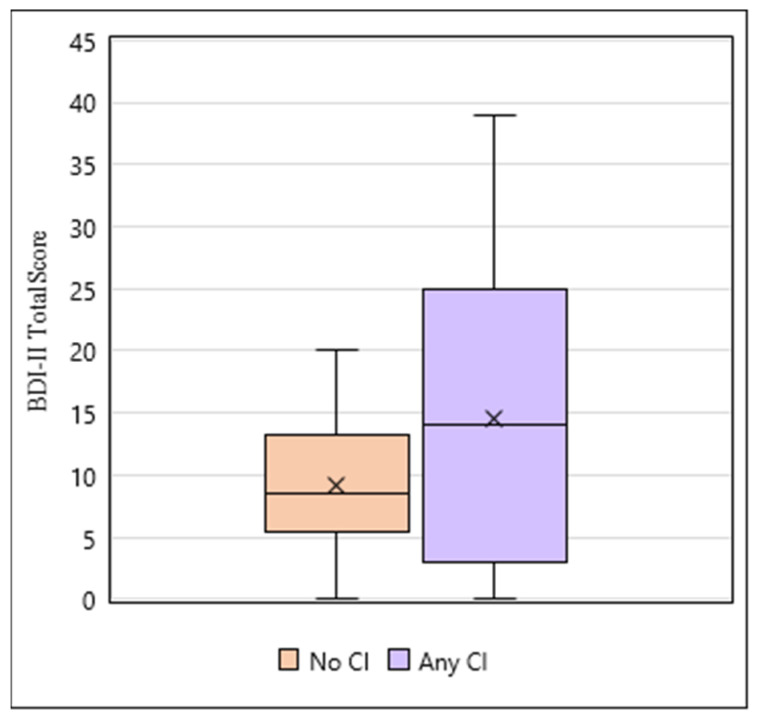
Association between depression and participants with no CI (n = 18) and any CI (n = 19). No significant difference in BDI-II score was found between the no CI vs. any CI groups (9.17 vs. 14.5, *p *= 0.099).

The association between depression and cognition in the study participants was also evaluated (Figure 4, Supplementary Figure S3). The median score on the BDI-II was 9 (IQR 5.75,19). BDI-II scores ≥ 14, indicating a positive screen for depression, were present in 40.9% (18/44) of participants. Moreover, 18.2% (8/44) had mild depression (score 14–19), 18.2% (8/44) had moderate depression (score 20–28), and 4.5% (2/44) had severe depression (score 29–63). The median score on the cognitive/affective scale was 5.5 (IQR 2.75, 11.25), and the median on the somatic scale was 4 (IQR 2.75, 7.25). The mean contribution of somatic symptoms to the total score was 41%.

There was no significant difference in mean BDI-II score between participants with no cognitive impairment and those with any level of cognitive impairment (9.17 vs. 14.5, *p *= 0.099). However, those with no cognitive impairment had a significantly lower mean BDI-II total score compared to those in the mild cognitive impairment group (9.17 vs. 19.4, *p *= 0.009), but no significant differences in mean BDI-II score were found between the no cognitive impairment and major cognitive impairment groups (9.17 vs. 10.1, *p *= 0.746) as well as between the mild and major cognitive impairment groups (19.4 vs. 10.1, *p *= 0.100).

## 4. Discussion

**Figure 5:**
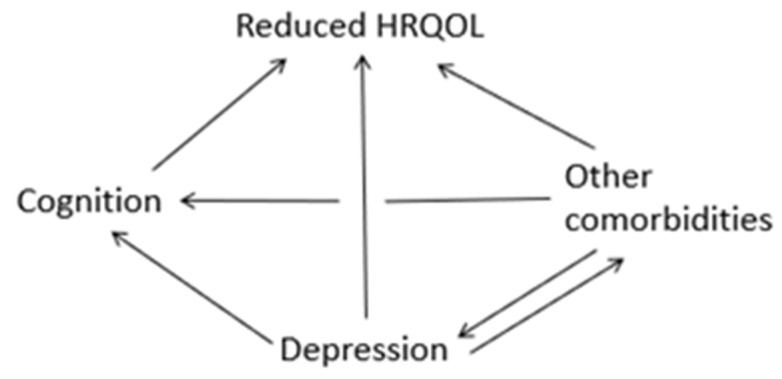
Proposed associations between depression, cognitive functioning, health-related quality of life, and other comorbidities.

Over the past several decades, iTTP has evolved from an acute fatal condition to a chronic relapsing disorder with long-term sequelae due to the availability of highly effective therapies for acute iTTP episodes. Previous studies have demonstrated a higher prevalence of cognitive impairment among iTTP survivors following recovery, reporting that 63% to 88% of study participants performed below expectations compared to controls [9,19]. iTTP survivors also experience reduced HRQoL and depressive symptoms over long-term follow-up. However, the association of depression and HRQoL with impaired cognition in iTTP has not been thoroughly explored. Evaluation of these associations between depression, cognitive functioning, health-related quality of life, and other comorbidities may provide insight into the long-term survivorship of iTTP survivors following recovery (Figure 5).

In this analysis, we show that reduced cognitive performance is associated with reduced HRQoL, specifically physical health-related quality, in iTTP survivors, which may warrant more routine and thorough cognitive screening as well as novel therapeutic and interventional targets to improve quality of life. It is also possible that common underlying factors, such as cardiovascular disease, stroke, autoimmune diseases, and pre-existing psychiatric comorbidities, contribute to these outcomes. Several patients report an overall reduced ability to work and return to normalcy, and further research is required to identify modifiable risk factors.

iTTP survivors are at risk of chronic neurocognitive deficits and cerebrovascular disease. Cataland et al. previously reported that up to 63% of iTTP survivors had some form of cognitive impairment, and 39% of participants from this same cohort exhibited ischemic changes on brain MRI [20]. Silent cerebral infarction is a risk factor for cognitive impairment [5] in the general population, and a recent study reported that silent cerebral infarction is associated with major cognitive impairment in iTTP survivors [21], which may impact the quality of life [22]. Among our participants, 53% had performed below the expected level in at least one of seven domains within the NIH cognition battery. We found that lower fluid cognition composite scores (which are expected to be impacted by acquired brain injury versus crystallized cognition composite that is generally preserved in acquired brain injury) are associated with worse scores on the PCS of the SF-36. If independently validated in larger studies, this association would provide an added rationale for interventions focused on reducing the incidence of sub-clinical neurologic injury and neurocognitive deficits.

While we identified an association between mild cognitive impairment and depression among our participants, we were unable to demonstrate a significant relationship between the BDI-II scores and cognitive impairment in our participants. This may be attributed to the smaller sample size. However, it is important to note that our survey confirmed that depressive symptoms are common after iTTP, occurring at much higher rates than the 4.7% to 11% depression prevalence in the general U.S. population [23,24,25]. Given the high proportion of women and obesity among our study participants, it is possible that common underlying risk factors, such as female gender and obesity, further contributed to the increased observation of depressive symptoms observed among iTTP survivors [25,26]. Additionally, we have seen that those with mild cognitive impairment may be at a higher risk of experiencing depressive symptoms, and routine depression screening and cognitive evaluation should be conducted in patients following up after TTP recovery.

The observation that 40% of participants self-reported depressive symptoms is indicative of the burden of mental health faced by survivors of iTTP. Pre-existing psychiatric diagnoses of depression, anxiety, and PTSD were reported by 32%, 21%, and 6% of study respondents, respectively, and may also have contributed to the decreased mental component scores observed in the HRQoL. Our group has previously shown that individuals with iTTP have high rates of PTSD associated with their diagnosis. Additionally, there is a higher burden of comorbidities, including SLE [27,28,29]. These underlying comorbidities likely contribute to impaired HRQoL as well as depressive symptoms in individuals with iTTP, as in other populations [30]. Thus, multidisciplinary survivorship care to address cardiovascular risk and other comorbidities prevalent in patients with iTTP [30]. Thus, multidisciplinary survivorship care to address cardiovascular risk and other comorbidities prevalent in patients with iTTP, as well as screening for and treating depression, may improve patients’ quality of life.

One limitation of this study is the modest sample size since iTTP is an ultra-rare disorder. The quality of life and symptoms of depression prior to the iTTP episode are not definitively known. Participants were asked to report any history of depression or poor quality of life prior to the iTTP episode (s), but this may be subject to recall bias, interview bias, and survey non-response bias. Further, the participants were provided with a one-time survey, and the cross-sectional nature of this study does not allow us to track changes in quality of life, depression, and cognitive function in patients, and the causative agents for the participants’ mental health outcomes are undetermined.

A large subset of the participants completed the survey instruments during the first two years of the COVID-19 pandemic, which may have had an impact on study outcomes. Participants were also prompted to discuss their COVID-19 experiences and report whether their responses to the surveys were largely influenced by the COVID-19 pandemic. All participants reported that the content discussed during this study was more closely associated with TTP, given the chronic nature of the diagnosis. However, since this study relies on patient-reported outcomes, there is an opportunity for interview bias, patient recall bias, and survey non-response bias. Finally, while we show association, this study cannot establish a causative link between depression and cognitive impairments and HRQoL. Still, these are important outcomes that highlight patients’ experiences and identify a need to investigate risk factors and potential mitigation measures to improve HRQoL in this vulnerable population.

In conclusion, we report a high prevalence of cognitive impairment, depression, and reduced HRQoL among TTP survivors and demonstrate associations between impaired cognitive functioning and depression and reduced physical HRQoL. Given that iTTP patients have a known risk for psychological comorbidities, cognitive decline, and poorer quality of life, it is imperative to establish optimal screening strategies for depression and cognitive impairment and to identify interventions to improve outcomes. Whether addressing depression and cognitive impairment (and its underlying causes) will improve HRQoL needs to be evaluated in prospective interventional studies.

## Figures and Tables

**Table 1:** Baseline characteristics of study participants (N = 47).

Characteristic	
Age, years (median, IQR)	51 (39, 60)
Female sex (%)	76.6
Race (%)	
Black/African American	76.6
White	16.1
Other	4.3
No. of TTP episodes (median, IQR)	2 (1, 3.5)
Comorbidities (%)	
Hypertension	42.6
Stroke	19.1
Chronic Kidney Disease	8.5
Diabetes Mellitus II	14.9
Obesity (BMI > 30)	23.4
Systemic Lupus Erythematosus	19.1
Hyperlipidemia	19.1
Psychiatric Diagnoses (%)	
PTSD	6.4
Depression	31.9
Anxiety	21.3

**Table 2:** Mean SF-36 scores for study participants and the general population.

SF-36 Variable *		TTP Survivors (N = 47)	General Population (N = 2474)	*p*
Physical Components	Physical Functioning	68.6 ± 27.8	84.2 ± 23.3	<0.0001
	Role-limitation Physical	50.5 ± 41.9	80.9 ± 34.0	<0.0001
	Bodily Pain	70.2 ± 27.5	75.2 ± 23.7	0.1532
	General Health	50.2 ± 22.0	71.9 ± 20.3	<0.0001
Mental Components	Vitality	46.3 ± 23.0	60.9 ± 20.9	<0.0001
	Social Functioning	69.4 ± 26.4	83.3 ± 22.7	<0.0001
	Role-limitation Emotional	52.2 ± 40.2	81.3 ± 33.0	<0.0001
	Mental Health	65.3 ± 19.3	74.7 ± 18.1	0.0004
Summary Scores	Physical Component Summary	43.2 ± 11.7	50 ± 10.0	<0.0001
	Mental Component Summary	43.2 ± 12.5	50 ± 10.0	<0.0001

* All scores reported as mean ± standard deviation (S.D.).

## Data Availability

Please contact the corresponding author with requests for deidentified data. All data requests will be reviewed for feasibility and priority.

## Supplementary Materials

The following supporting information can be downloaded at: https://www.mdpi.com/article/10.3390/hematolrep17050051/s1, Figure S1: Comparison of SF-36 PCS scores among participants with no cognitive impairment (CI) (n = 19), mild CI (n = 10), and major CI (n = 11), Figure S2: Comparison of SF-36 MCS scores among participants with no CI (n = 19), mild CI (n = 10), and major CI (n = 11), Figure S3: Association between depression and participants with no CI (n = 18), mild CI (n = 9), and major CI (n = 10), Figure S4: Association between depression and HRQOL.
