# Supplementary material for: Association of Cognitive Impairment with Reduced Health-Related Quality of Life and Depression Among Survivors of Thrombotic Thrombocytopenic Purpura
Source: Hematol Rep. 2025 Sep 27;17(5):51. doi: 10.3390/hematolrep17050051 (PMC12564109; doi:10.3390/hematolrep17050051)
Supplement: Supplementary file 1 [file hematolrep-17-00051-s001.zip › hematolrep-3735473-supplementary.pdf]

# DATA SUPPLEMENT

# Association of Cognitive Impairment with Reduced Health-Related Quality of Life and Depression Among Survivors of Thrombotic Thrombocytopenic Purpura

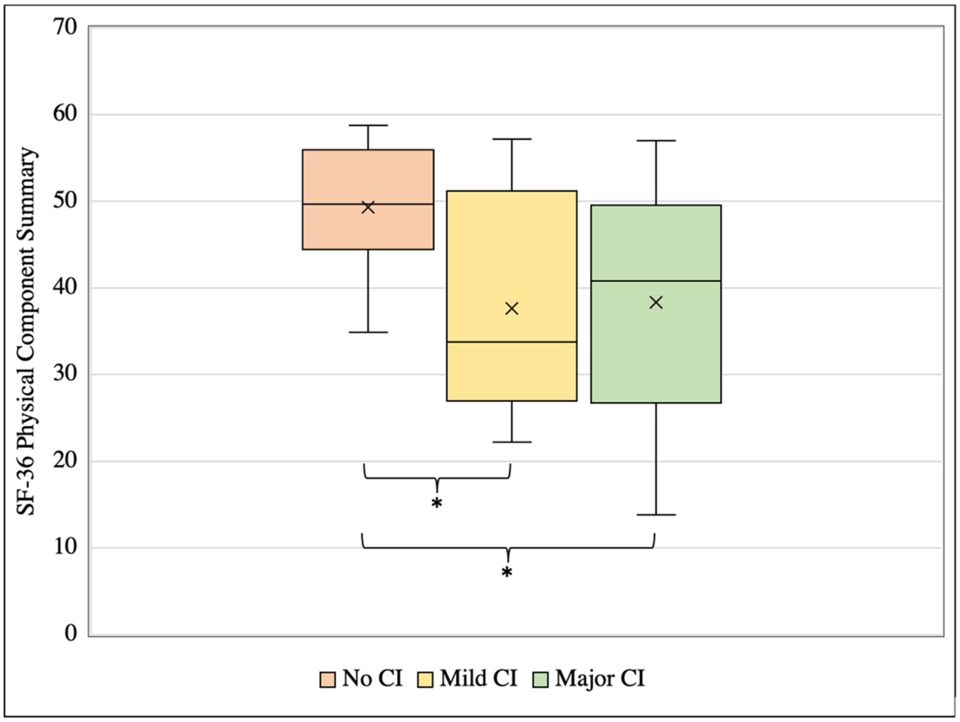


***Figure S1:*** Comparison of SF-36 PCS scores among participants with no cognitive impairment (CI) (n=19), mild CI (n=10), and major CI (n=11). The 'no CI' group has significantly improved SF-36 PCS scores compared to both the mild CI (49.3 vs. 37.7, p=0.005) and major CI (49.3 vs 38.4, p=0.007). No significant differences in PCS scores were found between the mild and major CI groups (37.7 vs. 38.4, p=0.906).

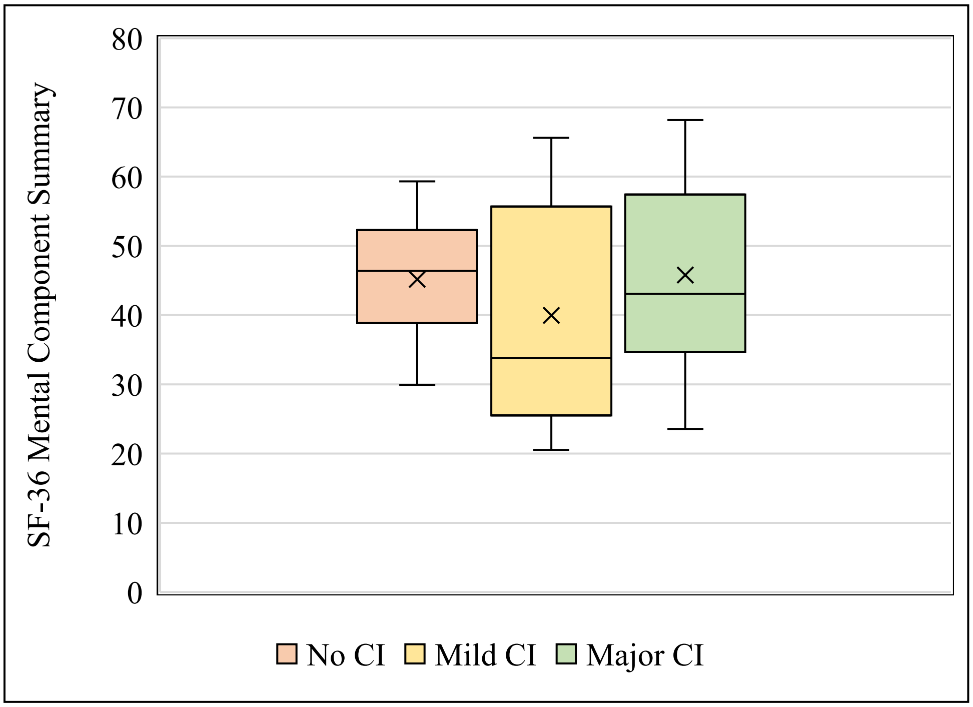


***Figure S2:*** Comparison of SF-36 MCS scores among participants with no CI (n=19), mild CI (n=10), and major CI (n=11). MCS scores did not differ significantly between the no CI vs mild CI (45.2 vs. 40.0, p=0.269), the no CI vs. major CI (45.2 vs. 45.8, p=0.880), and the mild CI vs major CI (40.0 vs. 45.8, p=0.385) groups.

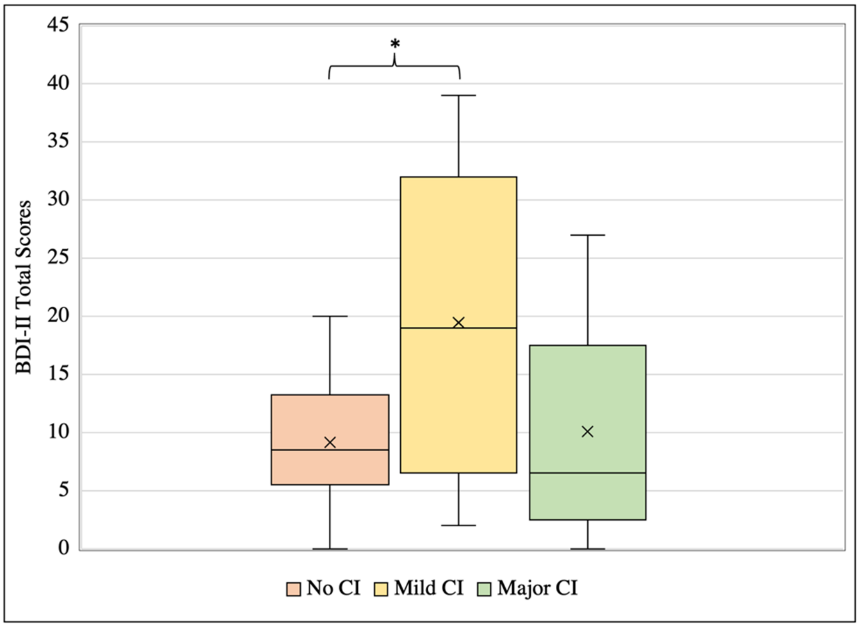


***Figure S3**:* Association between depression and participants with no CI (n=18), mild CI (n=9), and major CI (n=10). A significantly lower mean BDI-II total score was observed in the no CI group compared to the mild CI group (9.17 vs. 19.4, p=0.009). No significant difference in BDI-II score between the no CI vs. major CI groups (9.17 vs. 10.1, p=0.746) and the mild CI vs. major CI groups (19.4 vs. 10.1, p=0.100).

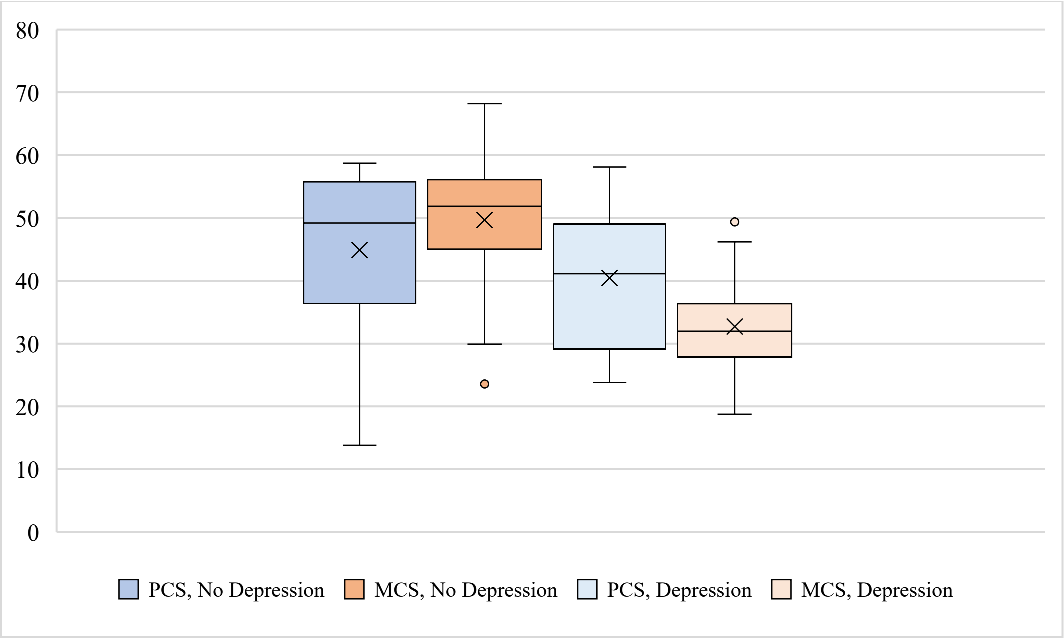


***Figure S4**:* Association between depression and HRQOL. Individuals with depression (BDI-II>13) had a significantly lower mean mental component summary score (p<0.0001) compared to those with no depression. The mean physical component score was not significantly different between the two groups (p=0.1799).
